# Disseminated fusariosis presenting as intracranial bleeding in a heart transplant recipient

**DOI:** 10.1128/asmcr.00056-26

**Published:** 2026-09-23

**Authors:** Nour El Meski, Praveen Gundelly, Sree Sarah Cherian

**Affiliations:** 1 Department of Infectious Diseases, University Hospitals Cleveland Medical Center 114516, Cleveland, Ohio, USA; 2 Clinical Microbiology, University Hospitals Cleveland Medical Center 114516, Cleveland, Ohio, USA; Pattern Bioscience, Austin, Texas, USA

**Keywords:** disseminated fusariosis, CNS fusariosis, intracranial bleeding, solid organ transplant, case report

## Abstract

**Background:**

Disseminated fusariosis is a serious fungal infection that primarily affects severely immunocompromised individuals. While it is well recognized in patients with hematologic malignancies and hematopoietic stem cell transplants (HSCT), it occurs far less frequently in solid organ transplant (SOT) recipients. Central nervous system (CNS) involvement is a rare clinical manifestation of disseminated fusariosis and has been scarcely reported in SOT recipients.

**Case Summary:**

We report the first case of disseminated fusariosis with CNS involvement manifesting as intracranial bleeding in a heart transplant recipient. This is a 48-year-old male with non-ischemic cardiomyopathy with reduced ejection fraction (<15%) and an implantable cardioverter-defibrillator (ICD) placed >10 years ago, admitted with cardiogenic shock status post impella device placement, followed by orthotopic heart transplant. Hospital stay was complicated by a spontaneous large intraparenchymal hemorrhage within the right parieto-occipital lobe measuring 4.7 × 3.3 × 4.8 cm on day 43 of admission, followed by acute respiratory arrest and hemodynamic instability on day 65 requiring intubation and vasopressors. Blood cultures and CSF cultures revealed *Fusarium falciforme*. A thorough skin examination revealed several dark lesions, mainly over his thighs bilaterally, along with bilateral toenail changes concerning for onychomycosis. The patient received treatment with intravenous amphotericin (10 mg/kg/day) and voriconazole (4 mg/kg twice daily), but he rapidly decompensated and passed away.

**Conclusion:**

While fusariosis is more prevalent among HSCT recipients, SOT patients remain at risk, and the outcomes of infection in this population can be equally devastating. Hence, early recognition and rapid initiation of antifungal therapy are crucial to improve patients’ outcomes.

## INTRODUCTION

*Fusarium* species are opportunistic fungi that are widely distributed in the environment. More than 50 species of *Fusarium* have been identified, including plant and animal pathogens (1). In humans, *Fusarium* can cause a broad spectrum of clinical manifestations, ranging from mild, superficial infections, such as keratitis, onychomycosis, and skin lesions (often in healthy individuals), to more severe forms where the infection becomes locally invasive or disseminated (particularly in immunocompromised individuals) (1). The clinical manifestations of fusariosis vary according to the site of initial infection and the host’s immune status, with immunocompromised patients being at increased risk of dissemination beyond the primary focus of infection. Disseminated fusariosis is a serious and often fatal manifestation of *Fusarium* infection in which the fungus spreads hematogenously to multiple organs. The CNS is a potential site of dissemination and typically occurs when the fungus spreads to the CNS through the bloodstream or by direct extension from nearby infected sites, such as the sinuses. Although rare, CNS fusariosis has been documented in a few cases in the literature; however, to date, it has never been reported as causing intracranial hemorrhage in a solid organ (heart) transplant recipient.

## CASE PRESENTATION

**Fig 1:**
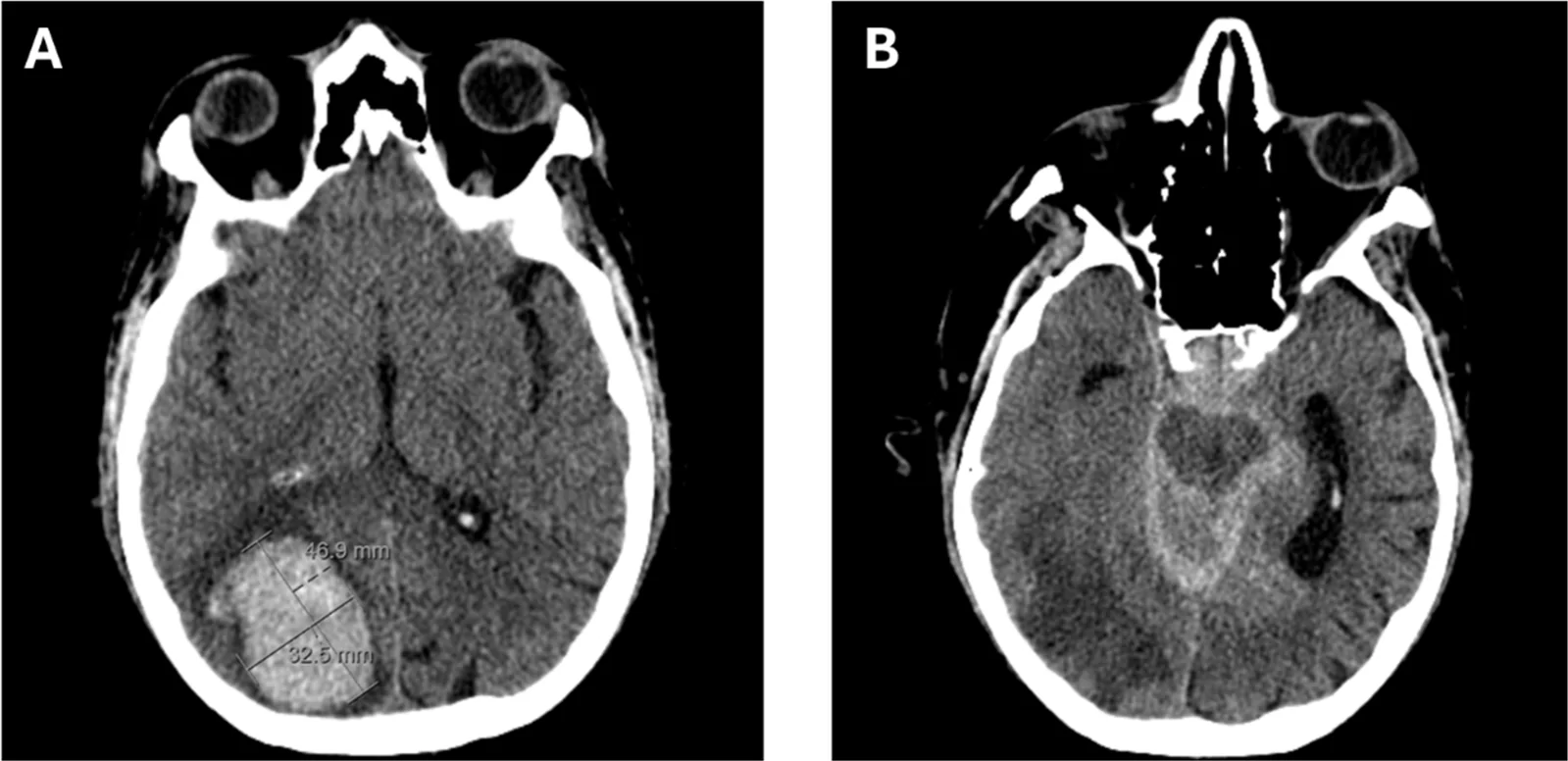
(**A**) Head computed tomography (CT) on day 43 of admission showing a large intraparenchymal hemorrhage within the right parieto-occipital lobe measuring 4.7 × 3.3 × 4.8 cm with surrounding vasogenic edema. (**B**) Repeat head CT on day 65 showing a new large amount of subarachnoid blood products within the basal cisterns, prepontine cistern, and within the folia of the cerebellum.

We report the case of a 48-year-old male with a past medical history pertinent for atrial fibrillation on apixaban, non-ischemic cardiomyopathy with reduced ejection fraction (<15%), and an ICD placed >10 years earlier, complicated by Group B *Streptococcus* bacteremia a year prior to presentation. The patient’s ICD pulse generator was ultimately removed, but the leads were retained due to high risk associated with surgical removal. The patient was referred for current admission following heart catheterization as an outpatient demonstrating cardiogenic shock. He had a prolonged hospitalization in the cardiac intensive care unit during which he underwent impella device placement on day 14 of admission, followed by orthotopic heart transplant (CMV D−/R−, EBV D+/R+, Toxoplasma D−/R+, basiliximab induction) on day 23. The organ donor was a young individual declared brain dead following a motor vehicle collision. Following transplant, the patient was maintained on tacrolimus 0.5 mg twice daily, mycophenolate mofetil 500 mg twice daily, steroid taper as per protocol, as well as voriconazole 200 mg twice daily, acyclovir 400 mg twice daily, and sulfamethoxazole/trimethoprim 400–80 mg once daily for infection prophylaxis. His hospital stay was complicated by ileus (Ogilvie syndrome), pneumonia, and acute renal failure requiring dialysis. On day 43, the patient developed a new onset headache with associated visual changes. Head computed tomography (CT) without contrast, followed by head CT angiography, showed a large intraparenchymal hemorrhage within the right parieto-occipital lobe measuring 4.7 × 3.3 × 4.8 cm with surrounding vasogenic edema (Fig. 1). Labs at the time showed a platelet count ~149 × 10^3^/uL (reference range 150–450 × 10^3^/uL), aPTT 24 s (reference 26–36 s), and normal INR. Potential etiologies for the intracerebral hemorrhage were thoroughly evaluated by the neurology team. Vascular causes, including underlying vascular malformations and vasospasm, were considered but not identified on diagnostic evaluation. The patient had a history of atrial fibrillation and had previously tolerated anticoagulation without prior bleeding complications, making anticoagulant-associated hemorrhage less likely. Additionally, the patient remained normotensive throughout hospitalization, making uncontrolled hypertension an unlikely explanation for the hemorrhage. Accordingly, his presentation was attributed to possible tacrolimus-associated posterior reversible encephalopathy syndrome (PRES), following which tacrolimus was stopped and switched to cyclosporine.

**Fig 2:**
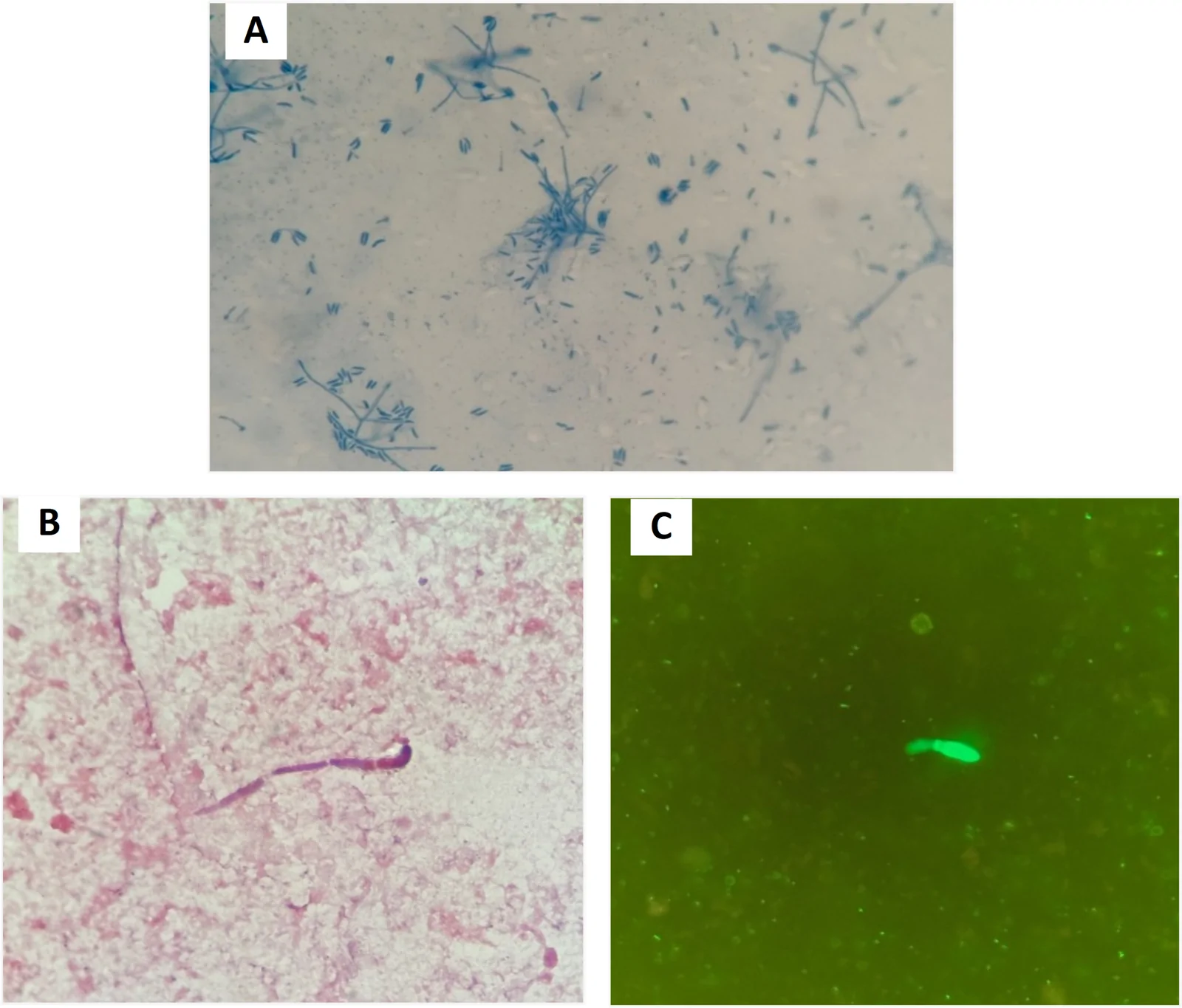
Pictures showing *Fusarium* elements growing on three different preparations from the positive blood cultures. (**A**) Lactophenol cotton blue (LPCB) mount. (**B**) Gram stain. (**C**) Calcofluor white stain.

**Fig 3:**
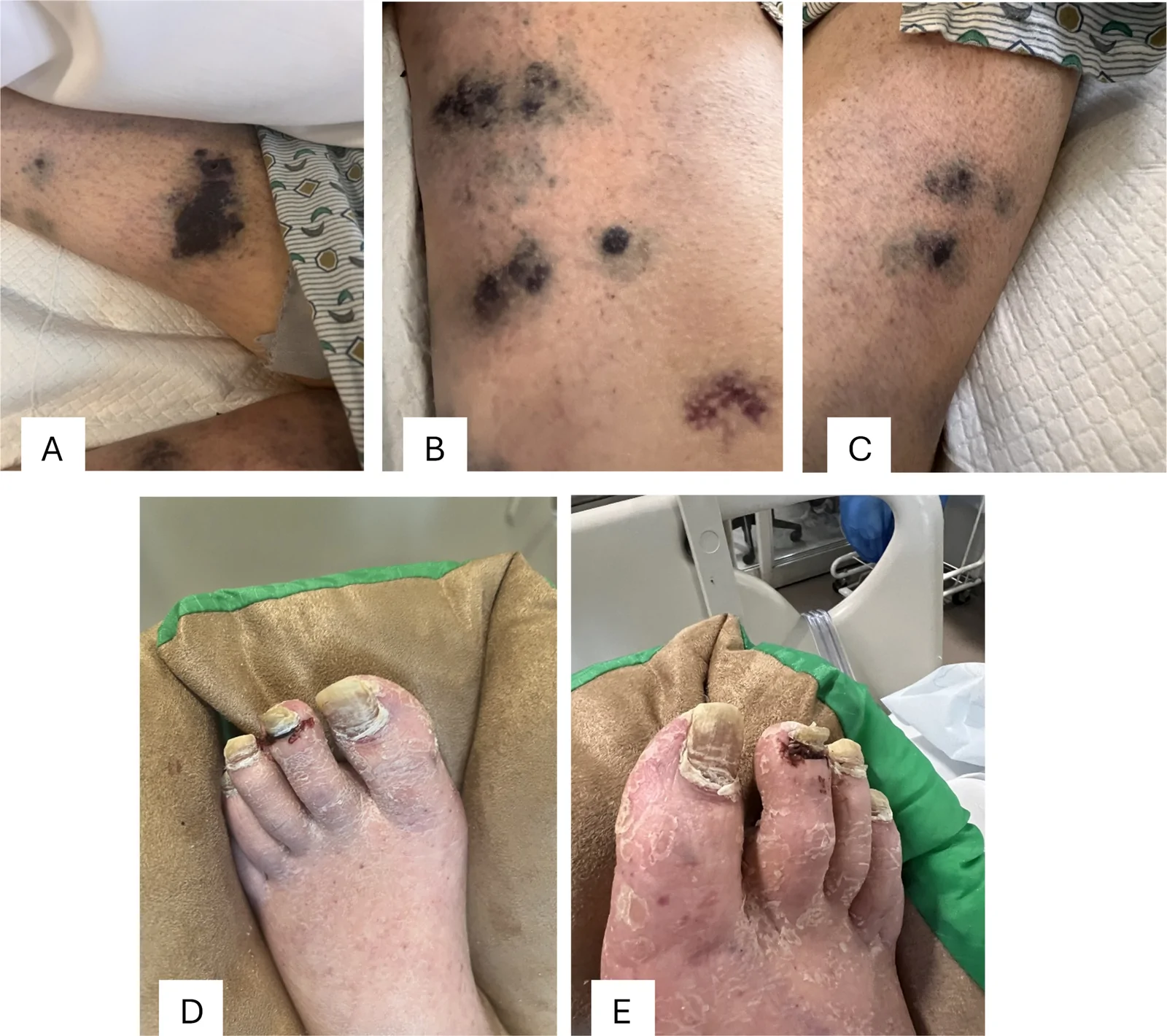
Dark-colored skin lesions over the patient’s arms and thighs, raising concern for possible *Fusarium* skin involvement (**A–C**). Bilateral toenail changes concerning for onychomycosis (**D and E**).

On day 65 of admission, the patient developed acute respiratory arrest and hemodynamic instability requiring intubation and dual vasopressors. He was started empirically on piperacillin/tazobactam 3.375 g every 6 h. Labs showed WBC 20.9 × 10^3^/uL (reference range 4.4–11.3 × 10^3^/uL) and platelets 124 × 10^3^/uL. Chest computed tomography showed overall improved multifocal consolidations as compared to prior, with residual consolidation primarily involving the left upper lobe. Head CT with contrast redemonstrated intraparenchymal right parieto-occipital lobe hematoma with interval increase in scattered supratentorial subarachnoid hemorrhage for which an extra-ventricular drain (EVD) was placed. An infectious workup was taken, including a CSF culture from his EVD. The patient has been afebrile otherwise throughout this period prior to and after his decompensation. His respiratory culture subsequently grew *Corynebacterium striatum*, for which vancomycin was initiated with renal dose adjustment. His blood cultures ultimately grew *Fusarium* species, initially detected in one of two sets on day 4 of incubation by the Roche cobas ePlex BCID Fungal Pathogen (BCID-FP) panel at our institution, with the second set turning positive on day 5. Accordingly, voriconazole level was ordered, and it resulted in 1.6 µg/mL (within normal range). Antifungal therapy was escalated with intravenous liposomal amphotericin B (5 mg/kg/day, increased to 10 mg/kg/day), while oral voriconazole was transitioned to intravenous therapy (4 mg/kg twice daily) (Fig. 2). At that time, the patient had an A-line, a right subclavian tunneled hemodialysis line, and a left femoral central venous catheter, which could not be removed given his hemodynamic instability and high pressor requirements. A thorough skin examination revealed several black lesions, mainly over his thighs bilaterally, along with bilateral toenail changes concerning for onychomycosis (Fig. 3). Unfortunately, the patient rapidly decompensated that day while on pressors before a dermatology assessment to rule out skin involvement or ophthalmology assessment to rule out endophthalmitis. A biopsy or culture was not attempted from skin and nail lesions, as he was transitioned to comfort care following discussion with his family members. He ultimately passed away the next day (day 71). Fungal stain of CSF showed the presence of hyphal elements. The CSF isolate was sent to a reference lab for further species-level identification and susceptibility testing and identified as *Fusarium falciforme*. The blood isolates were presumed to represent the same *Fusarium* species identified from the CSF. Antifungal susceptibility was performed, revealing a minimum inhibitory concentration (MIC) of 1.0 µg/mL for amphotericin B, MIC >16µg/mL for voriconazole, posaconazole, isavuconazole, and itraconazole, and MIC >64µg/mL for fluconazole (there are no CLSI breakpoints established for *Fusarium* species).

## DISCUSSION

*Fusarium falciforme* is a member of the *Fusarium solani* species complex (FSSC), which is responsible for the majority of *Fusarium* infections in humans (1). To our knowledge, this report illustrates the first case of disseminated fusariosis with CNS involvement presenting as intracranial hemorrhage in a heart transplant recipient. Previously, only one case of CNS fusariosis has been reported in a heart transplant recipient, in which dissemination manifested as endophthalmitis and intraparenchymal brain abscesses rather than hemorrhage (2).

The clinical manifestations of fusariosis vary according to the site of initial infection and the host’s immune status, with immunocompromised patients with hematologic malignancies, stem cell transplants, or profound neutropenia at increased risk for dissemination beyond the primary focus of infection (1, 3). Disseminated fusariosis often presents as a combination of cutaneous lesions and positive blood cultures, with or without involvement at other sites (the sinuses, lungs, joints, and others) (1). *Fusarium* species are angioinvasive and can produce circulating aleuroconidia by adventitious sporulation, making them more readily recoverable from blood cultures compared with other molds (4).

In many patients, skin lesions may be the first sign of a disseminated infection and are commonly seen in the early stages of the disease. Our patient did have concerning skin lesions, raising suspicion for skin involvement as well, but unfortunately, he rapidly deteriorated prior to a dermatology assessment.

Despite its higher frequency in patients with hematopoietic cell transplant (HCT) recipients, invasive fusariosis is uncommon in solid organ transplant (SOT) recipients. In a prospective survey among transplant recipients from 23 centers in the United States, 1,208 cases of invasive fungal disease in SOT recipients were diagnosed over a 5-year period, with only six cases caused by *Fusarium* species, underscoring how uncommon it is in this population (5).

Furthermore, among all clinical presentations, CNS fusariosis represents one of the rarest manifestations of disseminated fusariosis. A recent systematic review reported 26 cases of CNS fusariosis between 1974 and 2022, with varied clinical manifestations, including meningitis, leptomeningitis, meningoencephalitis, brain abscesses, cavernous thrombosis, and stroke (6). Among these patients, 60% had an underlying hematological malignancy, and 42% overall developed CNS fusariosis as part of their disseminated disease (6). Invasive fusariosis can lead to severe organ failure and has been associated with poor clinical outcomes and high mortality rates. Among the 26 patients with CNS fusariosis included in the systematic review mentioned above, the overall mortality rate was 72% (6). To date, only one case of CNS fusariosis has been reported in a heart transplant recipient, manifesting as a large 15 × 13 mm enhancing intraparenchymal lesion in the right frontoparietal region rather than intracerebral hemorrhage (2). No additional cases of CNS fusariosis have been reported, highlighting the rarity of this presentation and underscoring the need for further research in the SOT population. Interestingly, a case of *Fusarium falciforme* mediastinitis was recently documented in an orthotopic heart transplant recipient (7).

Patients with disseminated fusariosis typically exhibit a higher incidence of ophthalmologic manifestations, including bilateral endophthalmitis, compared with those affected by other CNS fungal infections (8). Unfortunately, our patient decompensated prior to an ophthalmology evaluation; thus, we could not rule out endophthalmitis.

Prompt initiation of an effective antifungal regimen and immunosuppression reduction are the cornerstones of fusariosis treatment. The treatment of fusariosis in general remains challenging, as *Fusarium* exhibits high MICs for commonly used antifungals (1). *Fusarium* species, particularly FSSC, may show elevated MICs to amphotericin B and triazoles, including voriconazole, posaconazole, and isavuconazole (1). However, reports in the literature provide conflicting evidence as to whether *in vitro* susceptibility data correlate well with clinical outcome (9). Current guidelines recommend high-dose liposomal amphotericin B or voriconazole as first-line therapy for invasive/disseminated fusariosis (10). Combination therapy has been described in several studies, as a potent alternative to antifungal monotherapy, especially in severe or refractory cases of fusariosis (11).

Despite progress in managing invasive fungal infections in recent decades, including the widespread use of antifungal prophylaxis in immunocompromised patients and improved therapeutic strategies, invasive fusariosis remains a serious and potentially life-threatening infection. While fusariosis is more prevalent among HSCT recipients, SOT patients remain at risk, and the outcomes of infection in this population can be equally devastating. Hence, early recognition and rapid initiation of antifungal therapy are crucial to improve patients’ outcomes.
